# Pulsation-related motion artifact of the external iliac artery mimicking localized dissection: An unexpected finding

**DOI:** 10.1016/j.radcr.2026.01.077

**Published:** 2026-02-13

**Authors:** Kanako Miyara, Nanae Tsuchiya, Takaaki Nagano, Satoko Yogi, Akihiro Nishie

**Affiliations:** a Department of Radiology, Nanbu Tokushukai Hospital. 171-1 Hokama, Yaese-cho, Shimajiri-gun, Okinawa, 901-0493, Japan; b Department of Radiology, Graduate School of Medical Science, University of the Ryukyus. 1076 Kiyuna, Ginowan-shi, Okinawa 901-2720, Japan; c Department of Thoracic and Cardiovascular Surgery, Graduate School of Medical Science, University of the Ryukyus, 1076 Kiyuna, Ginowan-shi, Okinawa 901-2720, Japan

**Keywords:** Aortic dissection, Motion artifacts, Arterial pulsation, External iliac artery

## Abstract

Contrast-enhanced CT is widely used for the evaluation of acute aortic dissection, but non–ECG-gated studies may still be affected by motion artifacts that mimic true vascular pathology. We report a woman in her forties who presented with right back pain. Non–ECG-gated contrast-enhanced CT demonstrated a discontinuous linear finding in the right external iliac artery, suggestive of a localized dissection. Follow-up CT performed 3 weeks later showed a similar appearance. Cine MRI subsequently revealed marked pulsatile motion of the right external iliac artery with approximately 6-mm displacement, resulting in compression and deformation of the adjacent external iliac vein. These findings confirmed that the CT abnormality represented a pulsation-induced motion artifact rather than true dissection. Although advances in CT technology have reduced motion artifacts, they may still occur in vessels with substantial pulsatile mobility. When such artifacts are suspected, complementary imaging—such as ultrasound, cine MRI, or ECG-gated CT—can facilitate accurate diagnosis and prevent unnecessary intervention.

## Introduction

Contrast-enhanced CT is a primary imaging modality for the evaluation of acute aortic dissection, and accurate interpretation is essential to avoid potentially catastrophic misdiagnosis. In non–ECG-gated CT studies, however, artifacts are commonly observed in the ascending and descending aorta due to respiratory motion, cardiac motion, and aortic pulsation, sometimes mimicking aortic pathology [[1], [2], [3], [4]]. In contrast, pulsation-related artifacts involving the pelvic arteries are exceedingly rare, likely because arterial motion in the pelvis is generally less pronounced than that of the thoracic aorta.

Here, we report a case of a pulsation-induced motion artifact of the external iliac artery on non–ECG-gated contrast-enhanced CT, which closely simulated a localized arterial dissection.

## Case report

**Fig. 1:**
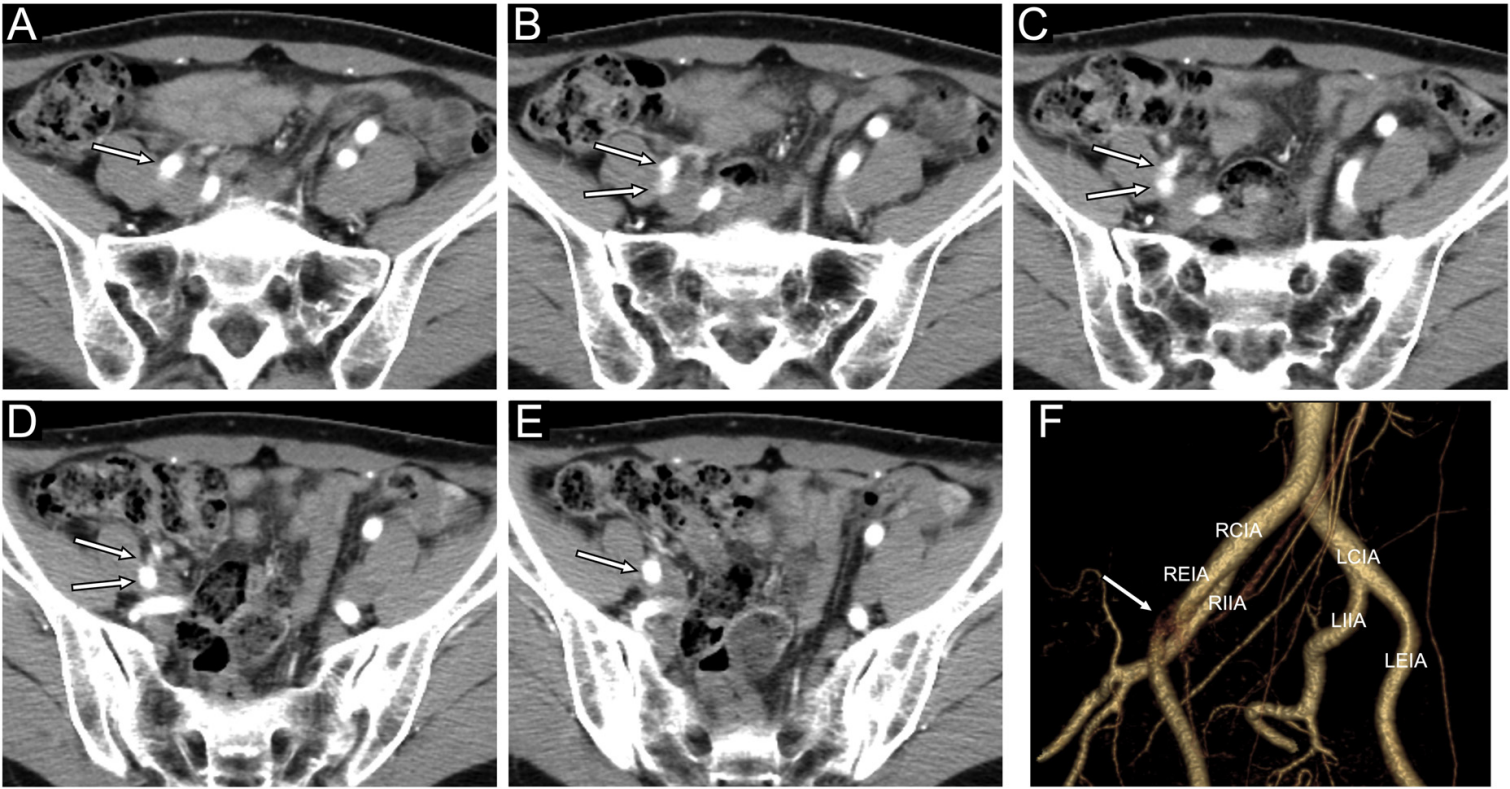
Continuous images of No-ECG gated contrast-enhanced CT (arterial phase, axial view). (A-E) and Volume-rendered images (right anterior oblique position). (F) showing discontinuous findings in the right external iliac artery (white arrows). RCIA, right common iliac artery; REIA, right external iliac artery; RIIA, right internal iliac artery; LCIA, left common iliac artery; LEIA, left external iliac artery; LIIA, left internal iliac artery. See also Supplementary Video 1.

**Fig. 2:**
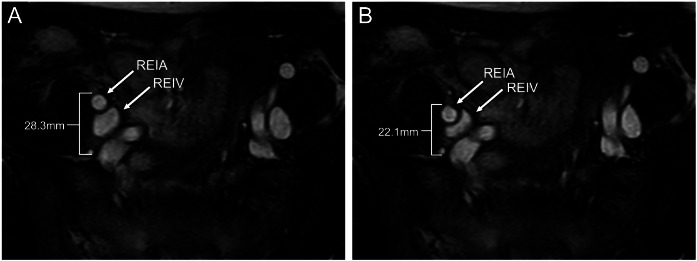
Cine MRI images (A, B) showing strong pulsation of the right external iliac artery, which displaced the artery by up to 6mm and compressed the adjacent external iliac vein. REIA, right external iliac artery; REIV, right external iliac vein. See also Supplementary Video 2.

A woman in her 40s presented to a local hospital with right back pain. Non-ECG-gated contrast-enhanced CT was performed, and discontinuous findings in the right external iliac artery were observed across the arterial, portal venous, and venous phases, raising suspicion of a localized dissection (Fig. 1). No discontinuous findings were observed in other arteries. Follow-up CT obtained 3 weeks later demonstrated similar findings, and she was referred to our hospital for further evaluation. Because the imaging findings suggested the possibility of artifact rather than true pathology, additional MRI was performed. Cine MRI revealed strong pulsation of the right external iliac artery, which compressed and deformed the adjacent external iliac vein, and showed that the artery moved approximately 6 mm with pulsation (Fig. 2). These findings established that the CT abnormalities represented motion artifact caused by arterial pulsation rather than dissection.

## Discussion

Previous studies have described several mechanisms responsible for arterial motion artifacts on CT. These artifacts are primarily attributed to cardiac pulsation, aortic wall motion, and the presence of highly attenuating adjacent structures, which can generate hypodense curvilinear interfaces or flap-like streaks mimicking aortic dissection. They typically occur in the aortic root and ascending aorta but may occasionally extend into the descending aorta [[1], [2], [3], [4]]. In contrast, motion artifacts caused by arterial pulsation in the pelvic arteries on contrast-enhanced CT are exceedingly rare.

With recent advances in CT technology—including shorter image acquisition times, improved reconstruction algorithms, and ECG-gated imaging—the frequency of vascular motion artifacts has markedly decreased [5]. Although artifacts due to suboptimal scanner performance were considered in this case, the imaging parameters met the recommended protocol for aortic CT angiography, and both scanning technique and CT system function were within standard specifications (64-row CT, beam pitch 0.516, gantry rotation time 0.4s, collimation 5 mm/0.6 mm, injection rate 2.9 mL/s, arterial phase at 33s). The examination was performed without ECG gating.

To our knowledge, only a single case of a pulsation-related motion artifact on non-ECG-gated contrast-enhanced CT has been reported in the left common iliac artery [6]. In that report, a similar artifact was also observed in the right external iliac artery of another patient, and in both instances, the artifacts appeared at markedly tortuous arterial segments [6]. In the present case, the artifact likewise corresponded to a curved portion of the artery.

Although the age of the patient with the right external iliac artifact was not specified, the patient with left common iliac involvement was in her thirties, and our patient was in her forties—thus within a similar age range. These patients were relatively young and presumably had minimal atherosclerotic burden. A previous study evaluating the relationship between arterial pulsation and atherosclerosis on contrast-enhanced CT reported that vessels with greater atherosclerotic stiffening were less susceptible to motion artifacts [7]. Accordingly, younger patients with more compliant arteries may exhibit greater pulsatile displacement and thus be more prone to motion-related artifacts.

When arterial motion artifact is unexpectedly suspected on CT, ultrasound may be helpful if the affected segment is accessible [6]. In addition, ECG-gated CT or cine MRI can minimize the influence of arterial pulsation and provide more accurate assessment [2,6].

In conclusion, awareness that pulsation-related motion artifacts can occur not only in thoracic but also in pelvic arteries is essential to avoid misdiagnosis. Such artifacts may be more frequent in younger patients with minimal atherosclerotic changes, likely due to greater arterial compliance and mobility. When an artifact is suspected, complementary imaging—including ultrasound, cine MRI for pulsatile evaluation, or ECG-gated CT to reduce motion—can be valuable for accurate diagnosis.

## Patient consent

Informed consent was obtained from the patient featured in this case report.

## Appendix Supplementary materials

Image, video 1 dummy alt text

Image, video 2 dummy alt text
